# Prognosis
1An address given to the meeting of the Bristol Medico-Chirurgical Society on February 13th, 1907.


**Published:** 1907-03

**Authors:** P. H. Pye-Smith

**Affiliations:** Consulting Physician to Guy's Hospital.


*"Scire est nescire, nisi id me*
*Scire alius sciret."*

MARCH, 1907.

### PROGNOSIS.[1]

BY

P. H. PYE-SMITH, M.D., F.R.C.P., F.R.S.,
*Consulting Physician to Guy's Hospital.*

PROGNOSIS is one of the most ancient branches of medicine. In early times pathology, or the science of disease, did not exist, and treatment was for the most part ineffectual as well as superstitious. But the forecasting of events, when based upon prolonged experience, justly impresses both the educated and the vulgar. We see this in the case of the oldest of sciences—astronomy, based on observations and proved by the power of prediction of eclipses. One can imagine how those who had scoffed at a mortal being able to foretell the overshadowing of the sun or moon, and to foretell it on a definite day or night, must have been impressed by the beginning and completion of the shadow of the earth. The same holds with other branches of science: the cultivation of crops and of flowers, construction of piers and bridges, the

[1] An address given to the meeting of the Bristol Medico-Chirurgical Society on February 13th, 1907.

power of transmitting messages by the electric telegraph, and the still more marvellous achievement of wireless telegraphy. Incredible as these discoveries once were they are no longer so, even when the explanation of the how and the why is incomplete.

So when a child is attacked by the symptoms of pyrexia it is a wonderful thing that we can foretell the progress of the disease, its gradual evolution, and, when the crisis is passed, its gradual return to the conditions of health. Nothing is more solid as a foundation for rational medicine than the power we have of foretelling within tolerably narrow limits the course of such diseases as syphilis, variola, and enteric fever. No justification can be more complete, no demonstration stronger to every candid observer, than the accomplishment of a prediction of a disease.

The following are a few examples from the prognosis of the father of medicine :—

He taught that when delirium disappears after the patient has slept he is likely to recover. That it is a bad sign for the convalescent to regain his appetite without putting on flesh. In cases of jaundice, if the liver becomes hard to the touch the prognosis is grave. When spitting of blood is followed by expectoration of matter the prognosis is unfavourable. When an abscess is opened by the knife or the cautery, if pure white matter flows out the patient will do well, but if it be mixed with blood and is foul in odour he will probably not recover. In acute diseases prognostics either of death or recovery are more uncertain than in those of a chronic course.

During the long retrogression which followed the Hippocratic era, when Galen taught anatomy on monkeys and hogs, and treatment was empirical and usually inefficient, the shrewdness and unbiased observations of the best physicians enabled them to form a prognosis, sometimes with remarkable success.

Again, next to immediate relief of pain there is no question so paramount with the patient himself as that of the event. What is it ? generally means, What will it do to me ? A favourable prognosis when given with authority is often more important than any method of treatment, and while we must never give patients hopes which cannot be fulfilled, we must preserve the priceless benefit of that determination to get well, of that interest in one's own case, which is one of the chief aids to recovery. The mere statement of the name of some obscure condition, and still more the prediction of recovery, however guarded as being probable rather than certain, will often ensure sleep in a despondent patient, and will rouse the effort which is needful for recovery.

The diseases of *children* afford very interesting examples of prognosis. In early childhood the patient can give us little or no account of his condition, and we have to depend entirely upon our own observation. Nothing, therefore, is better exercise for beginners than to investigate one of the exanthems or a severe injury in a little child. If they cannot help us by information they at least cannot set us on the wrong track (as adults frequently do), sometimes by wilful deception, but more often by the incurable mixing-up of facts and inferences and the obstinate refusal to tell us what no one else can—the patient's own feeling, and not a mixture of information from others, in supposed agreement with the theory of medicine.

In early life the processes of disease are very rapid, and a child may speedily be brought from the condition of blooming health to that of grave sickness, or even of imminent danger. But although this is true, particularly of diarrhœa and laryngitis, it is also true that children have a great power of resistance to a malady, so that his recuperative power not infrequently carries a child safely through extreme peril. Anatomical conditions of the child's body render the prognosis of invagination and laryngeal diphtheria peculiarly fatal; but diphtheria in children has been happily robbed of its terrors by the scientific treatment that has enabled us to meet the case with hope, and usually with success, which in patients under three years old was almost certain to end fatally.

When we deal with a disease of known pathology prognosis is comparatively easy. Cancer and hydrophobia, glanders and leprosy are soon recognised, and their diagnosis involves the gravity of the forecast. As soon as we have made the diagnosis of modified variola or of enteric fever we can predict the course of the disease and the order of its symptoms. When we have decided that a supposed scarlatina rash is a local erythema, or a scaly patch on the scalp is not ringworm, the prognosis carries with it the means of cure. Some of our elements of prognosis depend on the patient himself rather than on the disease. Certain symptoms, like hyperpyrexia, subsultus tendinum, or convulsions at once suggest a grave prognosis ; while, on the other hand, to see a patient's appetite returning, to observe his lying on his side instead of on his back, the diminished temperature and less frequent pulse, a recovery of strength and volume in the voice, are signs of improvement which we all recognise as guiding our prognosis.

Although their frame is fragile, we must all of us have been struck by the power of resistance of children in spite of loss of flesh, how they will rally after extreme emaciation or continued convulsions, or prolonged bronchitis. Even the worst forms of eczema are very seldom fatal. Children will struggle through rickets and will surmount the successive invasions of whooping-cough and bronchitis, of measles and diphtheria, scarlet fever and nephritis. Never give up a child till he is screwed down.

With older children, after the first dentition and until puberty is attained, the prognosis is much more favourable than in young children, and even in adults. Children between two and twelve are still subject to acute and severe illness, and may rapidly pass in a few hours from apparent health to imminent danger, but, compared with infants, their circulation is more stable, their vigour in breathing and coughing is much greater, and their power of maintaining temperature more perfect.

The older the patient attacked by diphtheria or scarlatina or ringworm the better is the prognosis. In one well-marked and severe disease—lobar pneumonia[1]—we must all have been struck by the wonderful power of recovery shown by children of between three and fifteen. We may say that death from uncomplicated acute pneumonia at this age is almost unknown. The same is true of enteric fever, the mildest forms of which are characteristic of this early age.

In this period, however, the prognosis of phthisis is very grave,

[1] Not "croupous pneumonia."

and it is often complicated by general disseminated tubercle. Scarlatina is more severe in these young subjects than in adult life, while measles is less so, and rubella is less severe and more safe at an older age.

There remains one of the most striking differences between the prognosis of a child and an adult—I mean *diabetes*. Happily rare in children and young adults, its danger is almost exactly measured by the age of the patient. The rare cases which occur in infants are probably always fatal, and the symptoms are very acute. In young adults it is still a very grave and menacing disease, and it is not till after forty that the cases become milder, less sudden in onset, and less severe in their symptoms, while, as we all know, diabetes in patients of advanced years is sometimes so mild that it is scarcely more than a bearable infirmity.

The period from twenty-five to fifty is probably on the whole the most favourable for diseases, acute or chronic. Cases of unvaccinated small-pox, if they recover at all, belong to early life, ten to twenty. During the period of forty to sixty, cases of phthisis are generally more chronic and more amenable to treatment than before. The muscular powers and the vigour of the heart, the respiration, and the brain are in most persons still at their best. When those who have attained an older period of life may still show remarkable mental ability, their enduring power is in almost all cases much diminished. The old man who would guard his laurels must speak but seldom. On the other hand, old people are less plagued with the sick headaches, indigestion, and eczema of early life. They are more liable to cancer, particularly of the stomach and rectum, but, according to the experience of most of us, less so to sarcoma. As old age advances, it is remarkable how many men and women retain considerable powers of endurance. Many old men bear short sleep and diminished rations better than they did in the prime of life, and are sometimes less fatigued than those twenty years younger by a day's shooting on a warm September day or a night in a railway carriage. Specific fevers are very fatal in old age. At the same time acute pneumonia, though never the almost innocuous disease of youth, is far from always fatal. I imagine the not infrequent cases I

have seen of men and women of sixty-five, or even seventy, who recovered from pneumonia, must be explained by their being picked lives, that is to say, that they have surmounted so many assaults in their long life that they are no longer ready to surrender at the first summons. Cancer is, as we all know, not infrequent in even the oldest, but it is in them that it is most often discovered for the first time after death. Very chronic cases of cardiac disease or Bright's disease are common in old age, but their symptoms are less severe than in younger patients.[1] In advanced old age, apart from these almost latent organic lesions, the chief mortality, like that of young children, is due to bronchitis or diarrhœa. Cerebral hemorrhage, though common in old age, is less so than at sixty; but we must remember in these, as in other cases, there are fewer persons living over seventy to furnish large numbers of any disease. In old people arterial disease is almost part of their natural condition, and probably there are few over fifty but have some loss of elasticity of their arterial coats. Hence the frequency of death in the aged, either from rupture of arteries in the brain or, still more suddenly, from syncope of the heart.

Lastly, I would urge the conviction that old age is not in itself a cause of death at all, however readily returns are made to the Registrar-General. I do not remember seeing a *post-mortem* examination in which there was not present degenerated blood-vessels, or renal disease, bronchitis, or diarrhœa, latent cancer, or some other obvious anatomical lesion.

Here let me urge the importance of drawing up the certificate of death either in accordance with an autopsy, or, when this cannot be obtained, in accordance with the symptoms during life. Apoplexy is a condition we can all recognise, and death from that cause is scientifically certain. But if we return "cerebral hemorrhage" as the cause of death, we are putting a probable conclusion only, and the result is that common diseases are made more common and rare diseases more rare than they truly are. Again and again I have seen such causes of death as anterior poliomyelitis, cancer of the pancreas, or tumour of the lung reported

[1] Mental and moral mellowing of old age—

"The soul's dark cottage, battered and decayed,
Lets in new light thro' chinks that time has made."

without any anatomical evidence. "Congestion" of the lungs or liver or other organs is another source of false facts. If active, the hyperæmia is only part of a local inflammation; if passive, it is due to some other primary cause. No doubt the double return asked for invites such secondary causes of death, but we are not obliged to make the double return, and a statement that a patient died of pneumonia, of phthisis, of renal dropsy, or of cancer of the liver is less, not more, valuable if bronchitis or pleurisy are added, or if we return cancer of the stomach instead of cancer of the liver, because we know that with few exceptions hepatic cancer is secondary to that of the stomach or bowels.

The difference between the sexes in the incidence of disease is much less than the difference of age. More girls than boys are subjects of chorea and whooping-cough. The exanthems affect both sexes equally. The frequency of scarlatina as a complication of delivery has long been recognised.

Speaking generally, better prognosis may be given in cases of serious disease when the patient is a woman than when a man. This applies strongly to gastric ulcer, and not to duodenal; also to valvular disease of the heart and to anæmia, but it certainly does not apply to phthisis.

In the large group of nervous affections we may, as a rule, give a better prognosis in the case of women. They more often recover from what has the appearance and many of the symptoms of organic disease: a cerebral hemorrhage, a tumour of the brain, acute myelitis or sclerosis of the spinal cord.

An important point in forming a prognosis is the different gravity of an acute attack occurring in a healthy organism and in a patient already suffering from chronic disease. Acute pneumonia, acute bronchitis, and infective fevers are all more grave in the case of patients already suffering from chronic disorders; and we may affirm that this is particularly true of diseases of the kidneys, the heart, and the lungs. The presence of Bright's disease is an important element in prognosis, not only when complicated by a new disorder, but when tried by injuries, including surgical operations, and by drugs like opium and mercury. Cardiac disease increases the mortality of bronchitis, of pulmonary catarrh (so-called " lobular pneumonia "), and acute lobar pneumonia. The presence of chronic phthisis and emphysema is most important in cases of acute bronchitis, whereas I have seen patients even at an advanced stage of phthisis go favourably through an attack of scarlatina, enterica, and acute rheumatism. Phthisis scarcely increases the danger of valvular disease. On the contrary, when cardiac incompetence is established it often appears to check the progress of consumption.

The presence of *glycosuria* has a marked deleterious effect on the prognosis of injuries or surgical operations, and also upon cases of acute pneumonia.

*Anæmia* differs so much in its causes and pathology that it is difficult to speak of its influence on prognosis generally. We may say that anæmia caused by direct hemorrhage, by injury or rupture of a blood-vessel, is less serious than when pallor appears during convalescence from fever or rheumatism, or when caused by privation or by deficiency of light and air.

The influence of *malaria* as an element of prognosis, like that of syphilis, has probably been overrated. It is very doubtful if *syphilis* is responsible for anything but its own direct effects; but we must include among the later effects of lues the chronic nervous degenerations of the nervous centres which have long been clinically recognised as general paralysis and tabes.

Another disease to which some writers attribute, I think, far too extensive an influence, is *gout*, particularly when the demonstrable anatomical effects are supplemented by various other symptoms which lead to drinking weak mineral waters and travelling to distant and expensive baths.

Prognosis of *enteric fever* depends, first, upon the age of the patient. In children it is as a rule a recoverable disease. I once saw a little boy pass through a severe attack in which there was every evidence of perforation of the bowel except seeing it, and he went out as healthy and much fatter than any boy of his age I have seen.

*Pneumonia*, like others of the group, is more favourable when primary, and less so when secondary to other diseases.

Many authorities, especially abroad, teach that *syphilis*, once acquired, is never got rid of, but therein I cannot agree. That it is a disease which often continues and relapses after apparent cure is a matter of common experience, but there are numerous cases in which all symptoms disappeared, the colour again became that of health, and the nourishment of the body as good as ever.

The prognosis of *phthisis* used to be considered almost hopeless, but at present it is generally recognised that the disease is curable. For proof of this we have only to frequent the dead-house of any large hospital to find in patients who have died from accidental injury, or some entirely different disease, unmistakable evidence of old phthisis in thickening and adhesions and contractions, so that the clinical course of the disease is confirmed by the results of *post-mortem* anatomy. We must all have seen such cases in practice, and noticed those who undoubtedly were affected in youth, living in health for many years, and dying at last from some other malady. This result is, however, far from constant. I have again and again observed that a patient who showed signs of tuberculosis in early adult life, after recovering and living for many years in health, may again develop symptoms of the disease of his youth. Two patients I have had for many years under observation in whom this result followed, and I am inclined to believe that the majority of cases called senile phthisis are really a recrudescence of a disease which was cured in early adult life.

*The prognosis of cardiac disease.*—In the early days of physical diagnosis it seems to have been thought by some of the leading physicians of the day that the presence of a murmur indicated incurable disease, and with the oracular solemnity of happily past times, an eminent physician, after listening to a patient's heart, said, "I am sorry to tell you, sir, that with the aid of this stethoscope I have heard your death-knell." Now valvular disease of the heart, regarded anatomically, is no doubt incurable, yet consequent changes in the muscular walls of the heart are sufficient to counteract the mischief done and to restore the hydraulic equilibrium of the blood. We hear such a process spoken of as an "effort of Nature" to deal with the disease. This is surely an inaccurate conception. There is no such thing as conscious or intelligent modification adapted to cure a disease. The "effort of Nature" in causing contraction of a serous surface sometimes remedies a dangerous lesion, but as often these same adhesions may cause fatal strangulation. The only healing power is the fact that any natural process, when not too violently disturbed, tends to return to its previous state of rest. The very processes which lead to a defence of the organism in one case will lead to its destruction in another. Even the phagocytes, which form such useful protection for an organism, certainly are not acquainted with the object of their life.

Passing now to the prognosis of *Bright's disease,* we may notice that an improved estimate of a patient's chances has replaced, as in the case of phthisis and of cardiac disease, the early estimates of its hopeless nature. The discoveries of Laennec, Corrigan, and Stokes, like those of Bright, were regarded as pathologically interesting, but as affording little room for treatment. Acute nephritis is sometimes, indeed, so rapid and severe—particularly in children—that bleeding, purging, and hot baths are powerless to avert the fatal event; but these cases are rare, and the ordinary forms of tubal nephritis are subacute in time and severity, and admit of ample time for treatment. In the advanced stages of the granular kidney treatment is much less efficacious; but even then it is astonishing how often a chronic case may be broken by gleams of hope, and the final cause of death be due, not to uræmia, but to cerebral hemorrhage.

In cases of ordinary cirrhosis of the liver, the organ is usually in a state like that of the corresponding disease of the kidneys, which depends upon a long course of toxic processes begun long before they come under notice. Quite apart from their direct effects, they probably cause still more numerous deaths when they act in greatly aggravating the prognosis of pneumonia or other acute attack.

Tuberculous meningitis is, with reason, regarded as of the worst possible prognosis, but I have seen it recover under the influence of mercury in cases which seemed as certain in their diagnosis as was possible without an autopsy.

Of the wonderful success in modern times of surgery in diseases of the brain we are all proud, and all the more because treatment is the result, not of chance good luck, but of carefully-reasoned scientific facts, acquired in the only way that facts in pathology can be discovered—by experiment on the lower animals. The diagnosis of a tumour of the brain, its accurate localisation and successful removal form, perhaps, the most brilliant example of surgery at its best which the past century offered.

I remember an observation which the late Mr. Stocker, the apothecary of Guy's Hospital in my student days, "obscurely wise and roughly kind," used to make in going round the wards to see urgent cases after the physician had gone home. "If," said he, "when you come with a candle to his bedside the patient lies indifferent when you pull down the clothes and examine the abdomen, that 's fever; but if he pulls them up again and turns over on his side, and swears at you for disturbing him, that 's brain."

The prognosis was often as sound as the diagnosis, but I am afraid in this, as in so many other matters, "the hits"—to use Bacon's simile—"are apt to be counted and the misses left out." The best prognosis, like the best diagnosis, rests on the foundation of observation and experience.

Of *gout and of rheumatism* it may be said that the arthritis they have in common seldom, if ever, leads to a fatal result. It is the rheumatic pyrexia which kills a patient with all the characters of a specific fever. On the other hand, gout has no such affinities, but proves fatal indirectly by setting up disease of the kidneys and the arteries. What our predecessors meant by gout in the stomach we do not know. We merely recognise it as an acute attack of flatulent dyspepsia, which can usually be cured by soda and ether with other anti-spasmodics. I have observed that the prognosis of rheumatic fever (provided that the heart escapes) is comparatively unaffected by anything but hyperpyrexia, and the joints seldom, and only after usually frequently-repeated attacks, become deformed.

*Osteoarthritis*, most unluckily still called rheumatic gout, though so distressing a crippling disease, has no internal allies, and is compatible with prolonged health for many years.

A fourth articular disease, also confused with gout and rheumatism, is gonorrhœal synovitis, remarkable by its origin, its distribution, and its relation to the eye, where it sometimes produces a subacute sclerotitis—quite distinct from infective iritis, due to actual contact with the streptococci of a primary focus—a true gonorrhœal ophthalmia. The prognosis of this rather uncommon affection is that it never attacks the heart; that though often obstinate and prolonged, it never returns when once got rid of, unless a fresh attack of gonorrhœa occurs, and then it almost always recurs, as bad as before.

The prognosis of what used to be called *typhlitis* or *perityphlitis* has changed remarkably in our lifetime. When described by Addison in 1850, it was generally cured by starvation or milk diet, opium, and local anodynes. The fatal cases were very rare. Now the same disease—for it must be the same—is often ushered in with great rapidity, and, unless early dealt with by the surgeon, is apt to lead to death. While admitting the frequency of such severe and rapid cases, I cannot throw away the experience of earlier days when typhlitis was a comparatively frequent disease, usually cured without operation, and only so treated when unusually severe or when the presence of pus was early recognised.

The prognosis of renal disease varies with age and circumstances. In children it is happily rare, though far from innocent. But in adults it is more manageable, and in old age a certain amount of albuminuria is compatible with comfort, and seldom ends in the rapid and profound coma seen in younger cases.

The prognosis of diabetes is better known now than it was in the days of Prout. In those under adult age it is rapidly and almost constantly fatal by coma. In early adult life it is a still dangerous disease. In old age it is comparatively free from danger.

Rational prognosis is the result of experience in cases, at the bedside and in the dead-house. If every organ could be the seat of every kind of disorder we should never come to a conclusion ; but we know that each organ has its own more or less restricted pathology, and therefore the path of accurate diagnosis and successful prognosis lies through the dead-house. On its portals might well be inscribed the sentence of our great master, Harvey : " Ad viliorum animalium inspectionem . . . accedite ; nam neque Dii desunt immortales maximusque omnipotens Pater in minimis."

May I conclude, as I began, with a few aphorisms of prognosis ?

Acute diseases following on chronic are the most dangerous.

A degree of pyrexia, which is unimportant in a child, is serious in an adult.

Typhus is most dangerous in the case of elderly patients and infants ; less so in young adults.

The most severe forms of scarlatina occur in children ; but whooping-cough is dangerous only in young children.

Pneumonia is benign, as a rule, in children and young adults, and is fatal in drunkards.

Acute primary pleurisy is only dangerous when the pericardium is also affected ; but the pleurisy secondary to tubercle, or to cancer, or to Bright's disease is usually fatal.

Œdema of the glottis is seldom the cause of death ; œdema of the lungs is often so.

" Capillary bronchitis " is in most cases lobular catarrh.

Hæmoptysis is seldom fatal by flooding the lungs. If it is, the cause is probably a small aneurysm of the pulmonary artery.

Hæmatemesis is seldom directly fatal. Valvular lesions appear to check rather than aggravate the progress of phthisis.

Sudden death is more often due to aortic incompetence than to aortic obstruction, and more often due to mitral obstruction than regurgitation.

Apoplexy, which is ingravescent, is almost always fatal.

Malignant growths are most rapidly fatal in children and young adults. In old age they spread slowly, and sometimes undergo more or less complete wasting.

